# Anti-Inflammatory Effect of Tacrolimus/Hydroxypropyl-β-Cyclodextrin Eye Drops in an Endotoxin-Induced Uveitis Model

**DOI:** 10.3390/pharmaceutics13101737

**Published:** 2021-10-19

**Authors:** Xurxo García-Otero, Cristina Mondelo-García, Francisco González, Roman Perez-Fernandez, Leandro Avila, Jose Ramón Antúnez-López, Miguel González-Barcia, Alfredo Adan, Pablo Aguiar, Francisco J. Otero-Espinar, Maria A. Bermúdez, Anxo Fernández-Ferreiro

**Affiliations:** 1 Pharmacology, Pharmacy and Pharmaceutical Technology Department, Faculty of Pharmacy, University of Santiago de Compostela (USC), 15705 Santiago de Compostela, Spain; xurxo.garcia@rai.usc.es; 2 Molecular Imaging Group, University Clinical Hospital, Health Research Institute of Santiago de Compostela (IDIS), 15706 Santiago de Compostela, Spain; pablo.aguiar.fernandez@sergas.es; 3 Pharmacy Department, University Clinical Hospital of Santiago de Compostela (SERGAS), 15706 Santiago de Compostela, Spain; cristina.mondelo.garcia@sergas.es (C.M.-G.); miguel.gonzalez.barcia@sergas.es (M.G.-B.); 4 Clinical Pharmacology Group, University Clinical Hospital, Health Research Institute of Santiago de Compostela (IDIS), 15706 Santiago de Compostela, Spain; 5 Surgery Department–CIMUS, University of Santiago de Compostela (USC), 15706 Santiago de Compostela, Spain; francisco.gonzalez@usc.es; 6 Ophthalmology Service, University Clinical Hospital, Health Research Institute of Santiago de Compostela (IDIS), 15782 Santiago de Compostela, Spain; 7 Department of Physiology, Center for Research in Molecular Medicine and Chronic Diseases (CIMUS), University of Santiago de Compostela, 15706 Santiago de Compostela, Spain; roman.perez.fernandez@usc.es (R.P.-F.); leandro.avila@usc.es (L.A.); 8 Pathological Anatomy Department, University Clinical Hospital of Santiago de Compostela (SERGAS), 15706 Santiago de Compostela, Spain; jose.ramon.antunez.lopez@sergas.es; 9 Department of Ophthalmology, Hospital Clinic of Barcelona, 08036 Barcelona, Spain; amadan@clinic.cat; 10 Paraquasil Group, Health Research Institute of Santiago de Compostela (IDIS), 15706 Santiago de Compostela, Spain

**Keywords:** endotoxin-induced uveitis, tacrolimus, eye drops, hydroxypropyl-β-cyclodextrin, interleukins, immunosuppressants

## Abstract

Background: Uveitis is an infrequent disease which constitutes a major cause of ocular morbidity. Correct management is essential, being corticosteroids its cornerstone. In case of contraindication to corticosteroids or treatment failure, the use of topical tacrolimus (TAC) could be an alternative which has already demonstrated safety and effectiveness in other ocular pathologies. However, TAC eye drops are not marketed, thus their elaboration must be carried out in Hospital Pharmacy Departments (HPDs). Methods: 32 Sprague-Dawley rats were divided into 4 groups of 8 rats each: (a) untreated healthy rats (Healthy); (b) untreated Endotoxin-Induced Uveitis model-rats (EIU); (c) EIU-rats treated with standard treatment of dexamethasone ophthalmic drops (DXM) and (d) EIU-rats treated with TAC-hydroxypropyl-β-cyclodextrin eye drops previously developed by our group (TAC-HPβCD). The mRNA expression levels of IL-6, IL-8, MIP-1α and TNF-α, quantitative analysis of leucocytes in aqueous humor and histological evaluation were performed. Results: TAC-HPβCD eye drops demonstrated to reduce ocular inflammation, expression of IL-6, TNF-α, MIP-1α and leukocyte infiltration in aqueous humor. Conclusions: TAC-HPβCD eye drops showed beneficial effect in EIU model in rats, positioning as an alternative for uveitis treatment in case of corticosteroids resistance or intolerance.

## 1. Introduction

Uveitis constitutes a heterogeneous group of intraocular inflammatory diseases which is defined as inflammation of the uveal tract that can secondarily affect adjacent structures, such as the retina or the optical nerve [1,2]. The overall prevalence and incidence of these conditions range from 5.81 to 19.42 per 10,000 subjects, and 2.51 to 11.12 per 10,000 person-years, respectively. [3,4]. Even though it is an infrequent disease, it constitutes a major cause of ocular morbidity, since it is the third cause of blindness in developed countries, representing an important health problem [5,6].

From the pathophysiological point of view, the immune system plays a fundamental role in the development of uveitis, whether or not it is secondary to infectious processes [7]. In this sense, the origin of inflammation can be attributed to an endogenous mechanism, either as part of autoimmune systemic diseases or as an isolated ocular condition [8]. Concerning the etiology of non-infectious uveitis, in recent years there have been significant advances in the understanding of their pathogenic mechanisms, pointing to the interaction between environmental factors and a complex genetic background, triggering a deregulated immune response able to overcome the ocular immune privilege [9,10]. In this regard, different proinflammatory molecules are increased depending on the specific origin of the disease [11,12]. In addition, several genetic determinants which might predispose to uveitis have been reported, either focusing on a particular disease [9,13,14], or analyzing the shared genomic basis, regardless the diagnosis [15].

Uveitis correct management is essential for preserving visual function and avoiding ocular and extra-ocular morbidity [16]. In this way, patients are usually treated based on the presence of signs of inflammatory activity, being corticosteroids its cornerstone, due to their immediate efficacy [17]. Location and severity of the inflammation will influence the change from topical to oral administration route. However, long-term administration of these agents can cause serious systemic and ocular adverse effects, such as hypertension, diabetes, and glaucoma [18]. In addition, when patients do not respond to this therapy or have a contraindication for the use of corticosteroids, it is necessary to resort to ophthalmic topical immunosuppressants in order to achieve a sustained control of the inflammatory process [17,19]. In this context, the use of topical tacrolimus (TAC) could be an alternative which has already demonstrated safety and effectiveness in other ocular pathologies including corneal graft rejection [20], vernal keratoconjunctivitis (VKC) [21,22,23,24], dry eye [25] or scleritis [26,27] among others. 

TAC is a calcineurin inhibitor with a potent immunosuppressive effect which has a mechanism of action similar to that of cyclosporine, but it is 10–50 times more potent, having demonstrated in clinical studies more effectiveness at lower concentrations and a better safety profile [19,28,29]. 

Regarding uveitis, TAC has showed a positive effect on the progression of the disease through reduction of inflammatory activity and suppression of the entire pathogenic pathway of the disease [28]. It inhibits the expression of Toll-like receptors (TLRs), macrophage inflammatory protein-1 alpha (MIP-1α), T-cell proliferation and the release of inflammatory cytokines such as interleukine-6 (IL-6), interleukine-8 (IL-8), and tumor necrosis factor alpha (TNFα), which have a key role in the development of uveitis [7,28,30,31,32]. Even though TAC has demonstrated to be effective in patients with refractory uveitis through oral [33] or intravitreal administration [34,35], topical application would be preferred because it avoids adverse effects related to systemic exposition to this drug and local complications after intraocular injections.

Despite all the above, the use of topical TAC for the treatment of uveitis has been limited because, nowadays TAC eye drops are not globally marketed. Consequently, this elaboration must be carried out in Hospital Pharmacy Departments (HPDs). Due to the deficient physicochemical properties of TAC, including low aqueous solubility and high molecular weight (804.02 g/mol), TAC eye drops must be prepared by reformulation from intravenous drug presentation (Prograf^®^) which contains ethanol and other compounds which usually cause discomfort and unpleasantness to patients [22].

In the last decade, several formulations of TAC for topical ophthalmic administration have been synthetized, including niosomes [36], nanovesicles [37], micelles [38] and nanocapsules [39]. However, it is not possible their translation to clinical practice because their synthesis is very complex to be carried out in HPDs. Most services do not have the necessary infrastructure to be able to develop these systems. The absence of specific equipment in production and quality control makes it impossible to produce these formulations optimally, and the personnel in charge of their production are not adequately trained to perform the necessary techniques in these processes.

In this sense, our group has previously developed and characterized a topical ophthalmic formulation of tacrolimus-hydroxypropyl-β-cyclodextrin (TAC-HPβCD) eye drops containing a 0.02% (*w*/*v*) of tacrolimus and a 40% (*w*/*v*) of HPβCD in Liquifilm^®^, demonstrating positive effects in terms of drug solubility, stability in aqueous solution and retention time on the ocular surface [40]. This formulation improves the currently prepared eyedrops in HPDs by replacing the irritating compounds of the intravenous drug presentation for HPβCD, which is the most appropriated cyclodextrin for topical ophthalmic administration according to the European Medicines Agency (EMA) [40,41].

The aim of this work is to demonstrate the anti-inflammatory effect of a TAC-HPβCD eye drops formulation, previously developed by our group, in an endotoxin-induced uveitis model in rats to provide for the first time a translational alternative for the elaboration of TAC eyedrops in HPDs.

## 2. Materials and Methods

Tacrolimus powder was acquired from Guinama^®^ S.L.U. (La Pobla de Vallbona, Spain), 2-hydroxypropyl-β-cyclodextrin Kleptose^®^ HPB (HPCD; MW = 1399 Da, substitution degree = 0.65 molar ratio) was provided from Roquette Laisa S.A.^®^ (Valencia, Spain), Liquifilm^®^ was purchased from Allergan^®^ Pharmaceuticals Ireland (Mayo, Ireland), Maxidex^®^ (1 mg/mL, eye drops) was purchased from Alcon Cusí, S.A.^®^ (Barcelona, Spain) and Lipopolysaccharide endotoxin (LPS; *Escherichia coli.*) was obtained from Sigma-Aldrich (San Louis, MO, USA). All other chemicals and reagents were of the highest purity grade commercially available.

### 2.1. Formulation Elaboration Procedure

The entire elaboration process of TAC-HPβCD ophthalmic eyedrops was carried out under sterile conditions in a flow laminar cabinet in the HPD of the University Clinical Hospital of Santiago de Compostela. First, the indicated proportion of hydroxypropyl-β-cyclodextrin (40% *w*/*v*) is dissolved in Liquifilm^®^ by stirring, when it was completely solubilized tacrolimus powder was added at a proportion of 0.02% (*w*/*v*) and left for 72 h under agitation (>750 rpm). Once this time has elapsed, the formulation is filtered through a 0.2 µm polyether sulfone (PES) filter (Stericup filtration system), and then packaged in sterilized polypropylene eyedropper bottles, showing to be stable for at least 3 months under refrigeration [40].

### 2.2. In Vivo Endotoxin-Induced Uveitis Model (EIU Model)

In vivo studies were carried out on male Sprague-Dawley rats with an average weight of 250 g supplied by the animal facility at the University of Santiago de Compostela (CEBEGA) (Santiago de Compostela, Spain). The animals were kept in cages under controlled temperature (22 ± 1 °C) and humidity (60 ± 5%), with day–night cycles regulated by artificial light (12/12 h) and feeding ad libitum. The animals were treated according to the ARVO statement for the use of animals in ophthalmic and vision research as well as the approved guidelines for laboratory animals [42,43].

The EIU was induced inoculating 1 mg/kg *Escherichia coli.* LPS diluted in 0.1 mL balance salt solution (BSS) into the rat’s right paw as previously described by other authors [44,45]. Once injected, the ocular inflammation peak reaches the maximum after 24 h [46]. 

**Figure 1:**
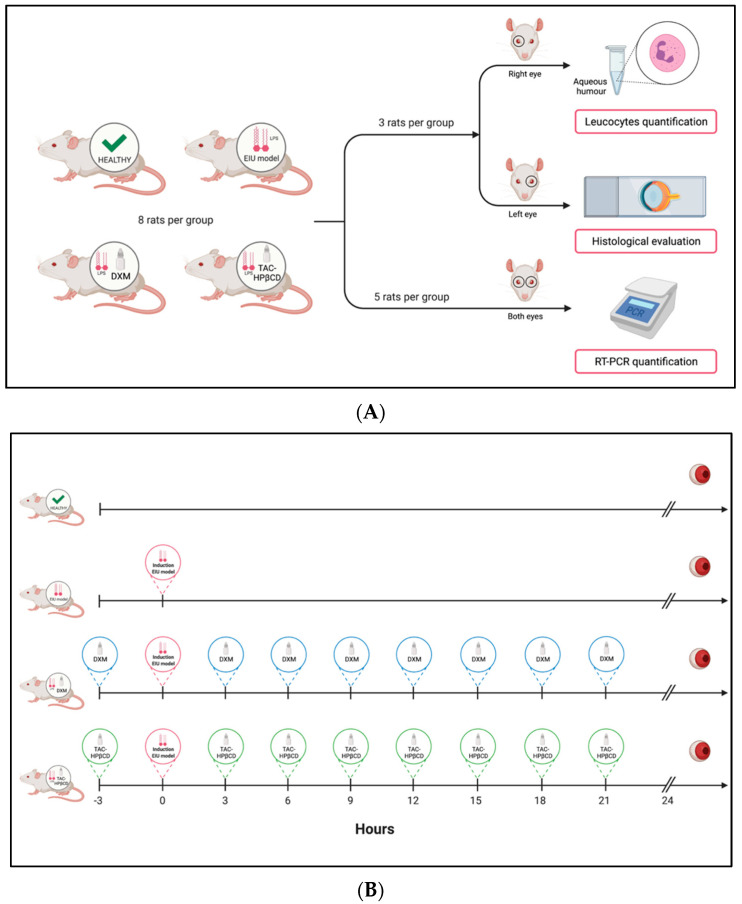
(**A**) Study groups scheme and distribution of eyes for their posterior analysis. (**B**) Study timeline: Induction EIU model, treatments, euthanasia and eyes removal. Created with BioRender.com.

In this case, 32 rats were divided into 4 groups of 8 rats each, as indicated in Figure 1. (a) untreated healthy rats (Healthy); (b) untreated EIU-rats (EIU); (c) EIU-rats treated with standard treatment of dexamethasone ophthalmic drops (DXM) (Maxidex^®^, dexamethasone 1 mg/mL, Alcon Cusí, Barcelona, Spain) and d) EIU-rats treated with TAC-HPβCD eye drops (TAC-HPβCD). Animals were anesthetized with 2.5% (*v*/*v*) isoflurane/oxygen and treatments were topically instilled (20 µL) on each eye 3 h before EIU model induction and every 3 h until 24 h later. Then, rats were euthanatized with carbon dioxide and all required samples were extracted.

### 2.3. RNA Isolation and Real-Time PCR Analysis

The mRNA expression levels of IL-6, IL-8, MIP-1α and TNF-α were evaluated in both eyes of the remaining 5 rats of each group by real time-PCR. After extraction, eyeballs were frozen at −20 °C and total RNA was isolated using TRIzol reagent (Invitrogen, Barcelona, Spain). Real-time PCR was made with 2 µg cDNA in a 20 µL volume using Luminaris Color HiGreenqPCR Master Mix (Fisher Scientific, Rockford, IL, USA). Samples were denatured at 94 °C for 10 s, annealed at 58 °C for 10 s and extended at 72 °C for 10 s for a total of 35 cycles. The amount of PCR products formed in each cycle was evaluated based on SYBR Green fluorescence with 18S as the endogenous control. Oligonucleotide sequences are detailed in Table 1.

### 2.4. Quantitative Analysis of Leucocytes in Aqueous Humor

For leucocyte determination, an expert ophthalmologist collected aqueous humor (10 µL) from the anterior chamber of the left eye of 3 rats from each group using a 28-gauge needle. Aqueous humor was stained (1:5) with Turk’s solution (Merk, Darmstadt, Germany) for leucocyte counting in a Neubauer chamber (Brand GMBH þ CO KG, Wertheim, Germany). Each aqueous humor sample was evaluated in triplicate. 

### 2.5. Histological Evaluation

For histological evaluation, right eyes of 3 rats of each group were fixed in 10% neutral buffered formalin for 24 h and embedded in paraffin routinely. Sections of 4 µm thick were stained with hematoxylin and eosin using a CoverStainer system (Dako-Agilent, Glostrup, Denmark). Six sections from each eye were examined to perform semiquantitative analysis by an anatomopathologist expert. Leucocytes surrounded ciliary processes were taken into account for counting and photographed using an Olympus BX51 microscope equipped with a MC170 camera (Leica, Wetzlar, Germany).

### 2.6. Statistical Analysis

Values were expressed as mean ± standard error of the mean (SEM) or standard deviation (SD). Average values were compared using one-way ANOVA with Tukey’s multiple comparison test. *p* values of less than 0.05 were considered statistically significant. Graph Pad Prism (Version 8.0.1) software (GraphPad Software, San Diego, CA, USA) was used for all calculations.

## 3. Results

### 3.1. Evaluation of the Effect of Tacrolimus on IL-6, IL-8, MIP-1α, and TNF-α

**Figure 2:**
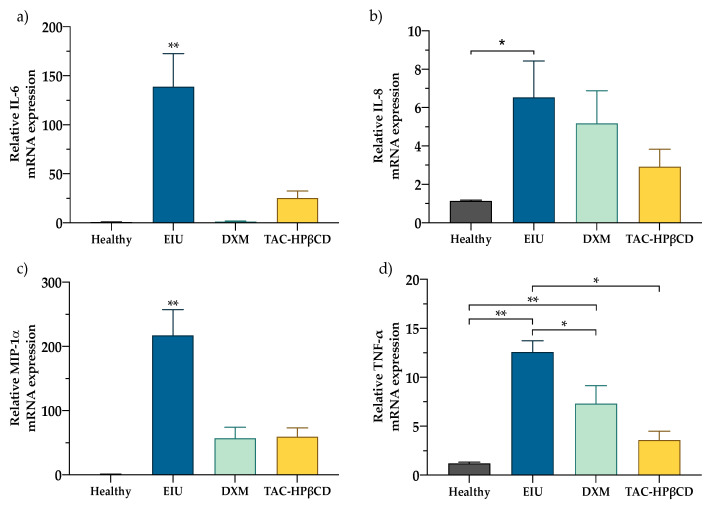
Anti-inflammatory in vivo effect (**a**) IL-6 (**b**) IL-8 (**c**) MIP-1α and (**d**) TNF-α mRNA expression in healthy eyes (Healthy), EIU untreated rats (EIU), EIU rats treated with dexamethasone (DXM) and EIU rats treated with TAC-HPβCD (TAC-HPβCD). (*n* = 10 eyes per group). Bars represent mean ± SD (* *p* < 0.05; ** *p* < 0.005).

The mRNA presence of the pro-inflammatory cytokines (IL-6, IL-8, MIP-1α, and TNF-α) in the eye tissues was assessed by real time-PCR in order to evaluate the effect of TAC-HPβCD on factors involved in the inflammatory process. The levels of cytokines in the eye tissues of healthy, EIU, DXM and TAC- HPβCD groups are shown in Figure 2 and the statistical significance of the different levels detected among groups is depicted in Table 2.

EIU group had considerable high levels of IL-6 (138.89 ± 89.71), IL-8 (6.53 ± 5.36), MIP-1α (217.37 ± 113.15), and TNF-α (12.58 ± 3.25) compared to healthy group [IL-6 (0.78 ± 0.68), IL-8 (1.14 ± 0.14), MIP-1α (1.20 ± 0.18) and TNF-α (1.22 ± 0.34)]. Consequently, the augmentation of all these factors was statistically significant in the untreated EIU group as compared with healthy rats, which means that the EIU model was properly induced. 

On the other hand, DXM group [IL-6 (1.29 ± 1.59), IL-8 (5.18 ± 5.09), MIP-1α (56.88 ± 54.70); TNF-α (7.31 ± 5.52)] and TAC-HPβCD group [IL-6 (28.69 ± 21.24); IL-8 (2.92 ± 2.73); MIP-1α (59.26 ± 41.43); TNF-α (3.57 ± 2.74)] had substantial fewer values of all the analyzed pro-inflammatory cytokines compared to EIU group. Hence, treatment with TAC-HPβCD and DMX significantly decreased levels of all these factors as compared with EIU group except for IL-8 whose reduction did not achieve statistical significance neither TAC-HPβCD group nor DMX.

Regarding the comparison of the detected levels of pro-inflammatory cytokines between healthy rats and TAC-HPβCD no statistically significant differences were found for IL-6, IL-8, MIP-1α levels, as well as between healthy rats and DMX group. This, along with the fact that no statistically significant differences were neither found among pro-inflammatory cytokines levels of TAC-HPβCD and DXM group demonstrate that TAC-HPβCD eye drops could be an alternative to topical corticosteroid therapy in the treatment of this model of EIU and their clinical translational use should be studied in future studies. In addition, it should be highlighted that with regard to TNF-α, statistically significant differences were detected between healthy rats and DXM group but no between healthy rats and TAC-HPβCD group. This fact suggest that TAC could be a better option than DXM when the uveitis is related with an elevation of TNF-α.

### 3.2. Quantitative Analysis of Leucocytes in Aqueous Humor

**Figure 3:**
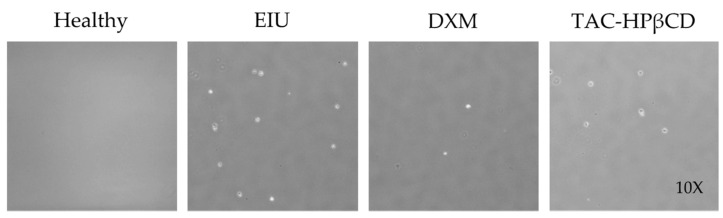
Leucocyte counting in aqueous humor 24 h after LPS injection in a Neubauer chamber. Groups were healthy eyes (Healthy), EIU untreated rats (EIU), EIU rats treated with dexamethasone (DXM) and EIU rats treated with TAC-HPβCD (TAC-HPβCD) (*n* = 3 eyes).

**Figure 4:**
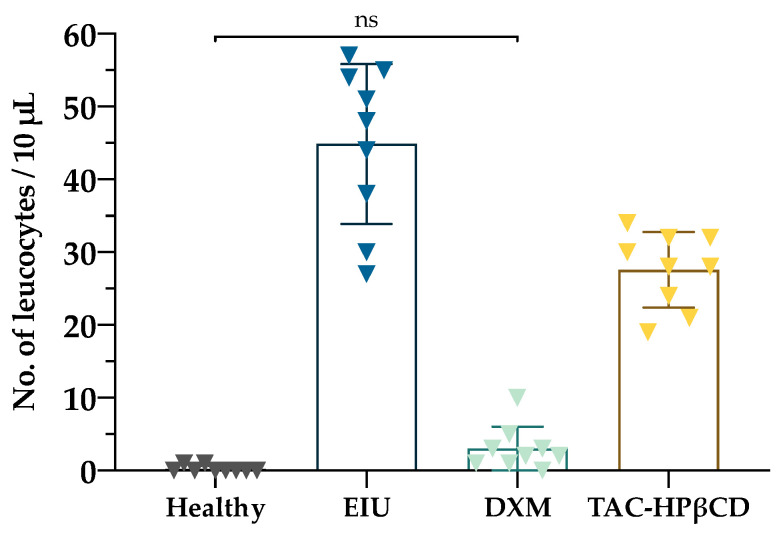
Quantitative analysis of leucocytes in aqueous humor. Groups were healthy eyes (Healthy), EIU untreated rats (EIU), EIU rats treated with dexamethasone (DXM) and EIU rats treated with TAC-HPβCD (TAC-HPβCD) (*n* = 3 eyes per group measured in triplicate). Bars represent mean ± SD. There are statistically significant differences among all groups except between Healthy and DXM group whose difference is non-significant (ns).

Leukocyte counts in aqueous humor of healthy, EIU, DXM and TAC-HPβCD groups are depicted in Figure 3 and Figure 4. In this sense, EIU group showed a significantly higher number of leukocytes (44.9 ± 11.0 cells/mL) compared to healthy group (0.2 ± 0.4 cells/mL) (*p* < 0.005), supporting the adequate development of the uveitis model. On the other hand, regarding leucocytes count of TAC-HPβCD group (27.55 ± 5.19 cells/mL), there is a considerable reduction in the number of leucocytes as compared with EIU group, being this difference statistically significant (*p* < 0.005) and positioning this therapy as a possible alternative to DXM treatment in uveitis. However, the decrease in the number of leucocytes is significantly greater in DXM group with regard to TAC-HPβCD group (*p* < 0.005), achieving a number of leucocytes comparable to healthy group.

### 3.3. Histological Evaluation

**Figure 5:**
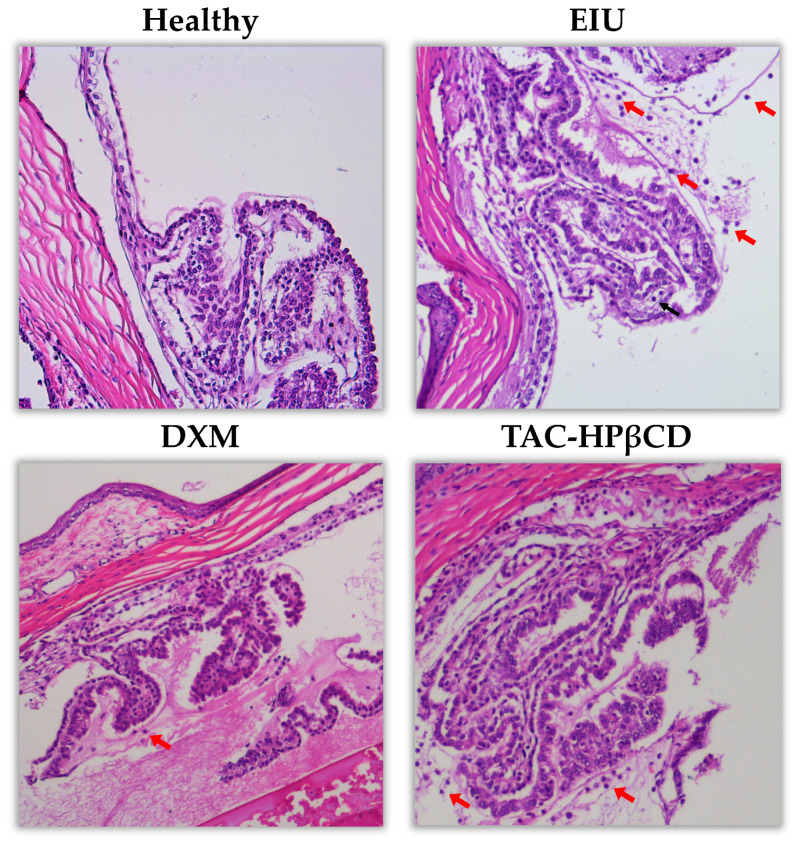
Representative hematoxylin-eosin staining showing infiltrated leucocytes (marked with arrows) in retina and ciliary processes of healthy, EIU, DXM and TAC-HPβCD groups. The histology study was carried out 24 h after LPS injection.

As it can be seen in Figure 5, the histological evaluation of retinas and ciliary processes showed no infiltration of leucocytes in healthy rats with no LPS injection. Very few or no leucocytes were found in DXM group while some leukocytes can be observed in TAC-HPβCD group. However, there was a considerable higher infiltration of leucocytes in untreated EIU-rats as compared with DXM and TAC-HPβCD groups.

## 4. Discussion

Nowadays, TAC is commercially available in oral and intravenous formulations, used in transplanted patients, and as an ointment for the treatment of atopic dermatitis [47]. TAC eyedrops can constitute an alternative in the treatment of uveitis [48,49] because it has been successfully used for various immune-mediated ocular disorders such as graft rejection [20], vernal keratoconjunctivitis (VKC) [21,22,23,24], dry eye [25] or scleritis [26,27]. However, TAC eyedrops are not marketed and they must be elaborated in HPDs. In this sense, it is necessary to highlight that TAC is a highly lipophilic macrolide lactone with a very poor water-solubility (1–2 μg/mL) [50]. This fact constitutes an important hurdle because TAC must be dissolved to allow efficient transport or permeation and reach the target site to become therapeutically effective [51]. 

Another inconvenience of TAC is that it has a high susceptibility to hydrolysis which leads to a very low stability in aqueous solutions [28,29]. For these reasons, TAC eye drops must be elaborated in HPDs by reformulation of the intravenous drug presentation (Prograf^®^), containing ethanol which have showed to disrupt the integrity of corneal epithelium and induce inflammation in corneal cells [52]. Based on this situation, in recent years several formulations of TAC for topical ophthalmic administration have been developed. In this sense, niosomes [29] and micelles [31] have been synthetized but they were not tested in vivo. However, positive effects of ophthalmic TAC were recently reported for nanovesicles [30] and nanocapsules [32].

Our group has previously developed a formulation of TAC-HPβCD eye drops containing a 0.02% (*w*/*v*) of TAC and a 40% (*w*/*v*) of HPβCD in Liquifilm^®^, which can be safely prepared in HPDs and showed to be stable for at least 3 months under refrigeration [40]. The major advantage of TAC-HβCD eye drops is that there is no need of using irritating excipients since our formulation strategy increases the solubilization of poorly soluble active ingredients and reduces the use of toxic products. In order to evaluate our formulation, a EIU model was selected because it is easily reproducible, quantifiable and clinical changes are similar to those of human acute uveitis [37]. Some authors stated that after LPS administration there is an activation of TLRs, increasing levels of proinflammatory mediators, developing inflammation of anterior uvea, choroid and retina and producing the breakdown of blood-humor barrier, with exudation of leukocytes into the aqueous humor [53,54]. In this sense, significantly higher levels of TNF-α, IL-6, IL-8, MIP-1α and leukocytes, as well as histological evaluation of EIU group, compared to healthy group confirmed the correct development of the uveitis model.

According to its mechanism of action, TAC inhibits calcineurin, blocks the production of proinflammatory cytokines and leads to inhibition of Th1 and Th2 cell activation [28,55]. In this regard, significantly lower levels of TNF-α, IL-6, MIP-1α and leukocytes, as well as histological evaluation of TAC-HPβCD compared to EIU group confirms the effectiveness of this formulation in the treatment of uveitis. Other authors stated that ophthalmic treatment with TAC solution in light mineral oil, a vehicle where this active substance is not adequately dissolved, was not able to improve any parameter related with clinical manifestation of uveitis comparing to a EIU model [37]. Consequently, our positive therapeutic effects using TAC-HPβCD demonstrate the proper solubilization of TAC in our formulation.

Similarly, TAC proglycosome nanovesicles were synthetized by Garg et al., showing promising results in a rabbit EIU model since they achieved suppression of expression of TNF-α and IL-6 in aqueous humor, and reduction in ocular inflammation, leukocyte infiltration and protein leakage into aqueous humor comparing with the administration of ophthalmic TAC solution [30]. Furthermore, Rebibo et al. elaborated TAC-loaded nanocapsules enabling a superior anti-inflammatory effect on an anterior chamber LPS-induced murine model of keratitis in comparison to the drug in oil solution and with a significant decrease in macrophage inflammatory protein 2 and IL-6, among other evaluated inflammatory markers [32].

Regarding the comparison between the efficacy of our formulation and the standard treatment with dexamethasone, no statistically significant differences were found among pro-inflammatory cytokines levels of TAC-HPβCD and DXM group demonstrating that TAC-HPβCD eye drops could be an alternative to topical corticosteroid therapy in the treatment of this model of EIU and their clinical translational use should be studied in future studies. It is necessary to highlight that Rebibo et al. and Garg et al., in contrast with us, did not compared their formulations with corticosteroid standard treatment [30,32]. 

In addition, concerning TNF-α mRNA expression, its reduction is statistically significant only between TAC-HPβCD and EIU group but no between DXM and EIU group, which is consistent with TAC mechanism of action [56] and could be especially beneficial in uveitis related to the augmentation of this factor, such as HLA-B27-associated uveitis, sarcoid uveitis and uveitis associated with Behcet’s disease among other noninfectious uveitis entities [11,12,57]. This possible personalization of the therapy based on the elevation of certain proinflammatory molecules could constitute an important advance, above all if this local increase could be correlated with systemic increases and detected through a blood test.

With respect to the limitations of our study, the fact that EIU model achieves the maximum inflammation 24 h after injection [36,37] makes necessary to carry out an accelerated dosage regimen and only permits a short-term evaluation which could constitute a hurdle regarding the translation of these results to the clinical setting. Another limitation is the lack of knowledge of the amount and rate of tacrolimus transcorneal permeation. Therefore, it will be necessary to study the intraocular pharmacokinetics of the TAC-HPβCD formulation with a suitable analytical method.

In spite of the fact that other ophthalmic TAC formulations have shown in vivo efficacy [37,39], as long as they are not commercialized, their translation to clinical practice is very difficult because of the high complexity of their synthesis. However, our TAC-HPβCD formulation has demonstrated in vivo efficacy and it can be easily elaborated in HPDs due to the simplicity of the elaboration process. Regarding the difference of cost associated between TAC eye drops usually prepared in HPDs by reformulation of Prograf^®^ and TAC-HPβCD eye drops, an estimate of the expenditure has been made in the Hospital Pharmacy Service for the two formulations in which the cost would be reduced by 200 euros for each patient and year with the implementation of the TAC-HPβCD formulation.

Future studies are needed to confirm the clinical effectiveness of TAC-HPβCD in order to consider this formulation as an alternative for the treatment of some forms of uveitis. The next step will be to conduct a study with patients in which the TAC-HPβCD formulation will be tested by comparing it with the results obtained in this group’s previous work [22] with the TAC formulation used in HPDs.

## 5. Conclusions

TAC-HPβCD eyedrops showed beneficial effect in EIU model in rats, thus they could be considered an alternative for uveitis treatment in case of corticosteroids resistance or intolerance. This formulation demonstrated to reduce ocular inflammation, expression of IL-6, TNF-α, MIP-1α and leukocyte infiltration in aqueous humor. In addition, the simple elaboration process of TAC-HPβCD makes possible to prepare this formulation in HDPs, improving in terms of safety the current elaborated TAC eyedrops by reformulation from intravenous drug presentation which contains ethanol and other irritating excipients. Further clinical studies are needed in order to evaluate long-term effectiveness of TAC-HPβCD.

## Figures and Tables

**Table 1:** Primer sets for real-time PCR.

Primers	Forward (5′-3′)	Reverse (5′-3′)
IL-6	CTTCAGGCCAAGTTCAGGAG	AGTGG ATCGTGGTCGTCTTC
IL-8	TCCAGCCAGTTGCCTTCTTG	GGTCTGTTGTGGGTGGTATCC
MIP-1α	CGTCCTCATCCTGATCACCT	GATACATCCCCGTGAACACC
TNF-α	CCAGATGGTCACCCTCAGAT	CCTTGACCG CTGAAGAGAAC
18S	GTAACCGCTTGAACCCCATT	CCATCCAATCGCTAGTAGCG

**Table 2:** Existence (YES) or absence (NO) of statistical significance calculated through a Tukey’s multiple comparison test among Healthy, EIU, DXM and TAC-HPβCD groups (* *p* < 0.05; ** *p* < 0.005).

Tukey’s Multiple Comparison Test	IL-6	IL-8	MIP-1α	TNF-α
Healthy vs. EIU	YES **	YES *	YES **	YES **
Healthy vs. TAC-HPβCD	NO	NO	NO	NO
Healthy vs. DXM	NO	NO	NO	YES **
EIU vs. TAC-HPβCD	YES **	NO	YES **	YES **
EIU vs. DXM	YES **	NO	YES **	YES *
TAC-HPβCD vs. DXM	NO	NO	NO	NO

## Data Availability

Not applicable.
